# Performing normality in working life among heavy substance users

**DOI:** 10.1177/14550725221108796

**Published:** 2022-06-28

**Authors:** Malin Gunnarsson, Jukka Törrönen

**Affiliations:** Department of Public Health Science, Centre for Social Research on Alcohol and Drugs, 7675 Stockholm University, Sweden; Department of Public Health Science, Centre for Social Research on Alcohol and Drugs, 7675 Stockholm University, Sweden

**Keywords:** embarrassment, face-work, Goffman, heavy substance use, life stories, normality, performance, stigma, work

## Abstract

**Aim:** Work is an important part of most people's everyday lives and well-being. Substance use by employees is associated with several negative consequences, such as absence from work and poor work performance. The study examines the strategies through which people who have problems with substance use produce a “normal” self and avoid becoming stigmatised in the workplace. **Methods:** The study uses data from in-depth unstructured life story interviews, which were conducted over phone with 13 people. The participants had developed various problematic heavy substance use habits. The interviews were analysed by applying interactional analysis and by using Goffman's concepts of “normality”, “embarrassment”, “face-work”, “stigma” and “performance”. **Results:** The analysis identified multiple strategies the participants used to produce normality and to avoid embarrassment and stigmatisation at work. These include skilful use of drugs in order not to show withdrawal symptoms, various ways of hiding their heavy substance use, frequent change of jobs, the maintenance of a clean and professional look, and attributing the absence from work to mental or physical illness. Moreover, the participants strategically avoided social contacts in which embarrassing situations could arise. When this was not possible, they manipulated their corporeal looks by hiding such kinds of bodily marks that would connote abnormality. **Conclusion:** The analysis points out that maintaining normality at work does not only refer to the efforts of trying to hide the effects of the drugs on behaviours and the body. It also reveals that the participants used substances to be able to perform energetically their work tasks, and in this way present themselves as normal workers. This ambivalence in performing normality makes the work life of people who use substances challenging.

Work is an important part of most people's everyday lives. In describing who they are, people often refer to what they do for a living. Work provides economic resources, an identity, a feeling of belonging, an opportunity to interact in social settings, and a choice to contribute to society in general. All these features have an impact on people's health and quality of life (Augutis et al., 2016; Wilcock, 2006). In addition, as adults spend most of their days at work, the workplace becomes a social context in which social norms about alcohol and other drug consumption are formed (Frone & Brown, 2010). In work-related literature, it is generally recognised that substance use has a negative impact on working life. For instance, a problematic use of alcohol and other drugs can make it more difficult to maintain full-time employment due to increased risk of absence or long-term sickness. Likewise, being present at work in an intoxicated or impaired state can impact work performance and increase the risk of errors (Buvik et al., 2018; De Sio et al., 2020; Godfrey & Parrott, 2005; Kemp & Neale, 2005). It may also increase the burden on colleagues who need to put in extra hours to finish the tasks heavy substance users are unable to carry out (Buvik et al., 2018).

While heavy substance use causes serious problems for working life, research also recognises that heavy users often try to continue working as long as possible since it makes their lives more meaningful, helps them to keep their daily routines and motivates them to maintain “normality” and represent themselves as responsible “good citizens” (Cebulla et al., 2004). Correspondingly, lack of employment may create a feeling of personal failure, resulting in experiences of shame and worthlessness (Robertson et al., 2021a, 2021b). In order to continue their working lives, heavy substance users may need to develop techniques to use substances when working. Otherwise they would not be able to do their work but would suffer from withdrawal symptoms, e.g., feeling “sick” or lacking energy (Cebulla et al., 2004). Being able to stay employed may also help people recovering from heavy substance use by encouraging them to maintain a regular daily rhythm, by increasing their social exchanges, and by strengthening their self-esteem even when they do not like their work (Pareaud et al., 2021; Sinakhone et al., 2017).

In this study, we draw on Goffman’s (1955, 1956, 1963, 1983) theories on face-work and stigmatisation to examine how people with heavy substance use produce the appearance of “normality” and avoid becoming stigmatised in the workplace through their self-presentations towards others. Prior literature suggests that Goffman's theories provide productive concepts to studying substance-use-related stigmatisation and face-work. According to these studies, stigmatisation is strongly present in the life of people who struggle with heavy substance use.

Heavy substance use often leads to a reduced capacity to work, economic complications, and difficulties being a functional family member, which increases substance users’ effort to maintain “normality” and portray a positive facade to others (Miczo, 2003). Wogen’s and Restrepo’s (2020) study shows that stigmatisation may affect care practitioners’ actions towards substance users and lead to unequal care and treatment. Juhila et al. (2020), in turn, demonstrate how people with heavy substance use and who need help from care workers with housing and the management of everyday life, do such kind of face-work towards care workers assign to help them, that may prevent them from getting the right form of care or treatment (Juhila et al., 2020). Many studies illustrate that heavy substance users tend to hide their problems by distancing themselves from stereotypical images of substance users (homeless, beggars, dirty) by avoiding or not participating in treatment and care (Hoolachan, 2020; Neale et al., 2011; Wogen & Restrepo, 2020).

To our knowledge, Goffman's dramaturgical theories on stigma and self-presentation have so far not been applied to examine how people who struggle with heavy substance use do face-work in the workplace to maintain “normality” in relation to others. In this study, by using unstructured life story interview data from a total of 35 participants, we address this knowledge gap.

## Approach

According to Goffman (1956), the concept of “normality” describes social interaction and encounters between people with a principle that any interacting individual has a moral right to be valued and treated in an expected, equal, and “normal” way. Performing “normality” in social interaction means being able to act in a given situation according to the expectations of others by following a standard of what normal behaviour in that specific situation means (Goffman, 1983). If an interacting participant is not able to convey an impression of “normality” but instead conveys an impression that contradicts or discredits the expected course of interaction, the situation may be experienced as confusing or embarrassing for all involved. The situation can feel wrong, and the participants may feel ashamed, embarrassed, or even hostile towards each other (Goffman, 1956).

Goffman stresses that embarrassment has a fundamental social and moral significance for moral order. It functions as a social mechanism that promotes mutual respect for interaction parties and makes the interaction orderly (Goffman, 1956; Schudson, 1984). He approaches this phenomenon through the concept of face-work, which emphasises the idea that people need to actively maintain a positive image of themselves and others and avoid losing face by controlling the impressions they give of themselves in encounters with others (Goffman, 1955; Mik-Meyer, 2020). People who are caught out in the act of telling a lie not only lose face in the moment but may spoil their identity for a longer time since people present can never really trust them again. It may also mean that people who have witnessed the lie no longer have a moral responsibility to take the liar's feelings into consideration in the future. Furthermore, if people feel that they have lost face they are likely to feel ashamed, inferior, or embarrassed because by their lie they have both done harm to themselves and have also threatened the security and self-images of the people present (Goffman, 1955, 1956). However, in everyday life people can use certain strategies to avoid putting themselves and others in face-threatening situations. For instance, they can use innuendo, be ambiguous, or maintain social distance. These strategies allow participants some space to build an impression of their own choice while at the same time protecting themselves from close inspection. Goffman calls these techniques “mystification” strategies (Goffman, 1955).

Goffman suggests that most social interactions and the strategies they involve take place in physical places which can be understood as “front stages”, which embody socially acceptable norms and values that are specific for each place. These “front stages” are critical for the performance of normality. In them people are expected to interact with others in an acceptable way (Goffman, 1956).

Moreover, Goffman's notion of stigma is important for our study. Goffman distinguished between three types of stigma: “abominations of the body” (such as deformities), “blemishes of individual character”, and “tribal stigma” (Goffman, 1963). In relation to this study, the first two are most relevant. Persons who become stigmatised may be viewed as not quite human, and their everyday life chances are then reduced by a variety of discrimination techniques. People experiencing stigma do not feel accepted or able to interact on equal grounds. They feel shame and typically respond to the situation by developing strategies (Goffman, 1963). The stigmatised persons may, for example, attempt to arrange life so that they avoid interaction with others, which can result in isolation, depression, hostility, or anxiety. When they need to interact with others, they may feel unsure of how these others will define them or what they really think about them. In such situations, the stigmatised persons may feel a need to overcompensate for their potential flaws by highlighting their “normality” or by diverting the focus from them on those with whom they interact (Goffman, 1963).

## Method and data

Life stories on heavy substance use provide rich research material, illustrating how substance use is related to various aspects of the narrators’ social encounters, areas of interest, and critical periods in the past. They show what kinds of events, encounters, and action have helped them to move on (Bengtsson & Anderson, 2020; Hänninen & Koski-Jännes, 1999; Miller, 2000). Life story interviews can also serve as a unique opportunity for the participants to reflect upon what has happened to them: what have been the most important events in their life and how the experiences, influences, and circumstances they have dealt with have affected them and made them the persons they now are (Atkinson, 1998; Freeman, 2006; Miller, 2000).

In this article we draw on life story interview data gathered for a larger project called “‘Addiction’ as a changing pattern of relationships: Comparing autobiographical narratives about different dependencies”. The larger project included 35 life stories with people who defined themselves as having a substance use problem. Some defined themselves as “addicts”, others emphasised having struggled at times with heavy substance use. The interviews were conducted by the first author. In 13 of the total 35 life stories on addiction, the narrators describe in detail how they used substances at work and what this meant for their performances of normality. Therefore, these 13 life stories were selected as the data for this article. These participants had different backgrounds in terms of social class, age, residency, and problem level. Among the 13 life stories, there were current substance users and previous users from different regions in Sweden.

All 13 interviews were conducted over the phone. We noticed that phone interviews facilitated the feeling of anonymity among the participants and increased their willingness to talk about sensitive subjects, such as heavy substance use. While conducting the interviews over the phone, we sensed that the participants felt more in control of the situation and more open to disclose intimate details about their lives than they would have been face to face, perhaps resulting in more honest, rich and in-depth data (Khalil et al., 2021; Novick, 2008; Trier-Bieniek, 2012).

The participants were recruited during the spring and summer of 2020 by purposive sampling. We used traditional recruitment techniques through social media by posting online a public advertisement on Facebook and Instagram. This proved to be an opportune way to recruit people who were not in treatment at the time. An informed consent was obtained before each interview. Each interview lasted about 50 minutes on average. All interviews were recorded. The participants were interviewed twice. The first interview was based on an unstructured interview technique. The participants were encourage to tell their life stories, including everything that happened between their first contact with alcohol and drugs and its later development until the present time. Having this kind of open approach ensured that the participant could focus on aspects that they themselves considered relevant and important for their substance use. During the interview, the interviewer remained a listener who sporadically asked exploratory and specifying questions that emerged in relation to what participants had told her about their lives (Brinkman, 2014).

In the second interview, the interviewer asked clarifying questions to map the nature of the social networks, settings, motivations, and consequences the participants’ substance use was related to. Each interview was fully transcribed, read carefully throughout, and then coded by using the NVivo software program.

The analysis proceeded so that first we coded the material by paying attention to the strategies and techniques the participants used to perform normality at work while using substances heavily. In this we drew on Goffman's concepts of normality, embarrassment, face-work, and stigma. In the coding process we noticed that the participants performed normality by using the following techniques and strategies: eliminating the withdrawal symptoms and producing normality by skilful and controlled use of substances, changing jobs frequently to avoid of losing face, maintaining a clean and professional look, hiding the substance use and bodily marks, avoiding social contacts, mystifying one's past and everyday life circumstances, and attributing absence from work to mental or physical illness.

Informed by this coding, we then selected three life stories for in-depth analysis. These life stories capture well the variety in the strategies and techniques of performing normality at work while using substances heavily and show what kind of contextual sensibility their use requires. Next, we analyse the life stories of Richard, Johanna, and Peter (the names have been changed).

## Analysis

In the analysis of how Richard, Johanna, and Peter produced normality at workplace while using substances heavily, we lean on Goffman's concepts as explained above. As our task here is to examine behaviour that is considered problematic or deviant, this makes the constructive nature of our participants’ social selves, activities and interaction visible for detailed analysis. In what follows, we focus on the strategies that Richard, Johanna, and Peter used to successfully produce normality at work but pay attention also to the situations and circumstances which made them lose control over their face-work and eventually cost them their job or changed the nature of their work.

## Results

### Hiding the effects of drug use, preventing withdrawal symptoms and changing workplaces

Richard is currently about 50 years old. His alcohol and drug use (cocaine and amphetamines) developed towards heavy consumption during his university studies. After graduation, he started working at different restaurants in the metropolitan area. Richard says that using alcohol and other drugs was common in his chosen profession, but there was an unwritten work rule within the trade that the use must not affect work or be seen by guests.

If we knew that there’d be a lot to do with service and if we’d been out the day before and were tired, then I could start my shift by taking [cocaine] to get started and make my head ready. Be a little more alert during the service, which could be maybe three to four hours. I tried to limit it so that it wouldn't be exaggerated. (…) I didn't want to show that I was intoxicated.

Richard developed various strategies to hide the effects of drug use and not to become too affected by it. Sometimes, if needed, he prepared himself for work using cocaine, but his preferred substance for this purpose was alcohol. Without drinking a bottle of wine before going to work he would not have been able to eliminate the withdrawal symptoms and act normally. Therefore, he took a “water bottle” filled with wine to work so that he could keep on drinking during work. Without continuous maintenance of the minimum level of alcohol in his blood, his impression management of normality would have collapsed and he would not have been able to carry out the required tasks:

I remember, I drank a bottle of wine in the morning and drove to work, filled my cup in moderation with alcohol to be able to cope with the day at work (…) It was usually every morning then, at eight or nine, when I got up if I went to work at ten o’clock. Always had a minimum of alcohol [in my blood] to be able to cope with my work, to talk, think, drive, or just to function at all.

Richard's occupation enabled him to move around a lot, which made it easier for him to hide his continuous use of alcohol and drugs. However, he needed to keep a close eye on the fact that his drug use was in constant danger of disclosure; he had to be ready to change jobs when he noticed signs of suspicion among his co-workers. Richard says that because he succeeded in building a rather good CV, he managed to switch from one workplace to another for many years. He kept on using drugs and whenever there were signs of “irritation” towards his substance use, he understood that he risked losing control and had to leave and start afresh at another job. The next quotation shows that the primary audience for his performances of normality was his boss, whose reactions he especially needed to monitor.

You felt it. That “well, okay, now I’ve annoyed my boss at my workplace enough, so now it's time to move on”. And I know that one reason why I was able to move so much and get new jobs was because I had worked up a pretty good CV. It was no problem to be able to do so [change jobs], all the time.

In addition, Richard made it sure that he looked professional in his work. He came to work clean in a well-kept uniform, which helped him to maintain the appearance of normality and to disguise the chaotic side of his life:

I was paid quite well and usually I had a uniform from work (…) and then I always went out [to the pub] in my uniform. I always worked in a suit, so we went out in the suit.

With the above strategies he was able to perform normality at work for about 15 years. Then his drinking and drug use had reached a level where he was no longer able to uphold the appearance of normal behaviour. His frequent job changes and irregularities at work had little by little contributed to stigmatising rumours, and there was no other solution for him than to move from the metropolitan area to a small town, where he managed to get a job in a less prestigious place.

The last day, when it really started to get out of my hands, I lived in [the metropolitan area] last time (…). The addiction took over so sharply then, so I started to have problems getting a job. All in all, I was blacklisted in [the metropolitan area]. People knew who I was, so it was difficult to hire me and that was when I made the decision to move to [a smaller city].

The move from the metropolitan area to work in a small town signalled for him the end of his ability to maintain the appearance of normality. After losing face, his addiction escalated further. He was no longer able to keep a job and he drifted into criminality and in part homelessness.

And at the same time, I had had new jobs that I got fired from all the time then, where I stole both alcohol and money. Lost control there. I kind of couldn't stop.

I made a brief attempt to work with someone else. But it didn't work out because I broke into her restaurant and took her alcohol. I also broke into someone else's [home] that I knew because I knew she had a safe at home, from which I took money.

### Using drugs at work, hiding injection marks and avoiding interaction with others

Johanna is in her late 20s. She started to use alcohol and marijuana as a teenager. After graduation she did her first detoxification and participated in the 12-step programme. This stopped her drinking but not her addiction that shifted to using amphetamines and pills. For a period, she became homeless before being admitted to treatment again. While she was recovering, she moved to another city and started to work in various temporary healthcare positions. She was not able to stay sober and started to use heroin.

Johanna's drug use caused a dilemma also known to Richard. To be able to keep up the appearance of normality at work, she needed drugs. Like Richard, Johanna developed various strategies to hide her substance use from patients and co-workers. Her strategies partly resemble and partly differ from those of Richard in producing normality. Like Richard, she developed techniques to control her withdrawal symptoms so that she could do her work in a normal, face-keeping way. She injected heroin every morning before going to work and also used it during the day. In comparison to Richard, who mostly used alcohol openly at work by pretending that he was drinking a non-alcoholic drink, disguising intravenous drug use was more of a challenge. Johanna describes how time-consuming it was to obtain the amount of illegal heroin she daily needed. Many times, the very arrangement of buying heroin was difficult, nor could she predict when it would happen. Therefore, she was often late for work or absent. If she could only buy heroin during the day, she misused sick leave.

There were days when I might not have gotten hold of the dealer at night, so I had to wait for the dealer so I could buy or take sick leave, because I couldn't go to work, I was too sick. [Or] I had to try to see the dealer while I was working.

Moreover, as she could not inject herself openly at work, she developed various excuses to go to the bathroom, such as complaining that she felt ill or had stomach ache. However, after a while these techniques lost their credibility because her colleagues started to wonder why she was spending so much time in the bathroom and what might be the cause of her health problems.

It works for a while, but it's damn difficult because you need to dodge in the bathroom, for example, at work. This takes time. And finally, colleagues begin to think about how bad your stomach really is. It was very, very difficult.

After coming to work, I went to the bathroom, took a fix, to make me abstinent. (…) As Suboxone contains naloxone, it was a total crash. So, I had to leave the bathroom and grinningly go to my coordinator and say that I felt dizzy, I needed to go home. And then I had been sitting on the toilet for maybe an hour.

Like Richard, Johanna also changed jobs frequently, which enabled her to better hide her drug use, because her co-workers did not have time to get to know her well enough and to figure out what she was doing. Moreover, in her temporary jobs it was common that there was no time for discussion with co-workers, which also made it easier to hide her consumption.

During the time I worked there, there were some very intense workdays. There was not much time to sit and talk. When you went to work, it was just full speed. And then it was lunch and then it was full speed [again], so if you wanted to talk, you did it in your leisure time or during lunch.

However, lunch breaks and free time posed face-work problems, providing a time and a place to get to know co-workers. Johanna therefore devised ways of not participating. When her co-workers asked her to join them, she explained that she had to be alone to recover from stress. By taking social distance and isolating herself, she avoided putting herself in a situation that could cause embarrassment, feelings of inferiority, or stigmatisation (Goffman, 1955).

During lunch when everyone wanted to gather and sit and talk, then I went (…) to another room to eat. I explained that I needed to be alone, to recover. So, I skipped [socialising] at lunch by isolating myself.

When she could not avoid talking with her co-workers, she used a tactic of saying something vague about her background or well-being and then quickly returned the question back to her interlocutor. She thus relied on a “mystification” strategy (Goffman, 1955) concerning her life and diverted focus to the lives of others.

People have always experienced me as very trustworthy, so I’ve been able to turn around [the conversation] very easily. If people start talking to me, I can say something superficial and turn around and ask: “But what about you?” And then the person can just tell me about it … about bad moods and crises and disasters … So, I’ve managed to stay away [from my personal issues] by turning everything back.

Like Richard, Johanna paid attention to her physical appearance and meticulously manipulated it to appear normal and avoid stigmatisation. To this end she dressed like everybody else and maintained good oral hygiene.

I can maintain a façade. I still have my own teeth … I don't look like a stereotypical addict. I look like everyone else and have [always] done and still do. It helps me.

However, it was much more challenging to cover the injection traces caused by heroin use. Johanna tried a cream that hid the injection marks, but this was not a guaranteed technique to produce the appearance of normality. Instead, she had to rely on her co-workers being casual, naïve, and inattentive.

In all care professions you should have short-sleeved shirts, but many [heroin users] inject … like me, I injected in my hands, chest, arms. I had to try to find a good cover cream, and it worked for a while … until I needed to wash my hands. And since there are a lot of double-manned shifts in the care service, I couldn't immediately go and put on a new layer of cream … it would raise suspicions to start smearing the hands with … I was a little lucky to end up in a working group that was quite relaxed and very naïve.

Like Richard, Johanna was able to hold her face of normality for several years. When Richard's performances of normality were especially attentive towards bosses, Johanna's primary audience and front stage consisted of co-workers. When preparing and carrying out her performance of normality, she approached intimate situations and interaction with co-workers as front stages. But one night she mixed heroin with benzodiazepines. She became self-destructive, injuring her head and face during the night. Instead of staying at home, she came to work six hours late in a confused state.

I’d start at nine in the morning and end at nine in the evening and it was very important that I arrived on time. I checked the clock at two o’clock at night. Then I had taken a lot of benzo and I sat all night on the toilet in the bathroom and hit my forehead on the edge of the bathtub. When I woke up and started getting dressed and going to work, my face looked like an alien. I was really bloated. I was completely unrecognisable. It looked damn bad. And my eyes, I couldn't even see them, because they were completely swollen. But I went to work. First, I was maybe six hours late. I had no idea. I came to work. And then I remember that I banged my head against the medicine cabinet inside some [patients’ room] and then a colleague came and said “I take over here”. I just “no, but you need …” I could hardly talk.

This incident which caused her to lose face was a turning point of her work career. The employer sent Johanna home, and her abnormal behaviour raised concerns that something was wrong. The employer requested a urine sample, which Johanna was not willing to take. She quit her job instead.

And then there was a nurse that I liked very much. She said “I have to talk to you” and then we went into [her office]. She said “how is it?” I said “it's great, just a little tired”. “A little. You’re going home right away”. “No, I can work”. “You go home at once, otherwise…” Yes, but… “we’ll call the police then”. She wasn't happy at all. I don't even remember if I changed [clothes]. (…) I emailed my boss to explain what happened. And then they demanded a urine sample. And I thought “no, but you’re an idiot, you can't do that”.

Johanna quit her job, but the damage had already been done. The way she left her work made her “lose face” and created a feeling of being embarrassed. Not only did this interaction negatively affect her management of her stigma in her current workplace, it also had a lasting effect. As she could no longer get a reference that enabled her to apply for a new job, she lost an important resource for the performance of “normality” in the labour market.

### Stabilising routines and performing a sober self

Peter is in his late 50s. He developed a habit of drinking as a teenager in his first job, where most people drank a lot of home-made beer. This wet manual labour environment led him to start drinking as well (Frone & Brown, 2010), and he quickly became addicted to alcohol. During weekends he drank so much that he was not able to come to work on Mondays and Tuesdays: he soon lost his ability to maintain the appearance of normality and was eventually fired.

Yes, a couple of times a week. I bought five-litre cans. I don't know, I just started knocking it back. It became an addiction right away. And then I couldn't do the job. I didn't have a full week in the two and a half years and then I was fired.

After various odd jobs and further education and training, he met a woman, got married, had children, and managed to find a permanent manual job. These changes in his life made him develop more stabilised routines and habits and balanced his drinking to the extent that he was able to come to work every day. He says that he became more responsible. But we can also interpret that the new circumstances pacified his lifestyle and enabled him to maintain better the appearance of normality. He was further guided to pay more attention to his impression management so that he could keep his job and provide for his family.

At that time at [the manual labour workplace], even if I drank, I always got up at half past six in the morning and [went to work]. I was always happy then. My girlfriend, she was very angry at me in the mornings because I walked around and whistled and so on. I don't know why [I went to work every morning]. It was probably that I wanted to take responsibility for myself. I mean, I was no more than 16, 17, 18 in my previous jobs … I was 20 years old then. Partly, I was going to be a father, and I liked my job. I became more responsible.

Even though he continued to drink daily, the stabilised circumstances helped him to keep his self-presentation in the sphere of normality, and he was able to maintain his job for many decades. Then came a divorce, and his alcohol consumption escalated. He started to drink at work too, and as he learnt that there was a worker who knew about his drinking but did not care about it, he planned his work shifts so that this person was his co-worker:

And then I had alcohol with me at work too. And the one I worked with, he knew about it. It became so that I planned who I worked with so I could drink at work. But he said nothing, but he knew I had a problem. I do not know why he did not reveal me.

On the other hand, like Richard, Peter tried to hide his drinking at work by pretending to drink non-alcoholic beverages and by using cough drops to cover the smell of alcohol.

Yes, it was in a plastic bottle and stuff. I had coffee with me. By then I had already mixed it [alcohol] in the thermos. And there was a lot of cough drops in all varieties. So, I always smelled like cough drops.

In this way, he did face-work not only to his co-workers but also to himself. He performed a sober self for himself so that he could feel belonging to the category of common human being.

Eventually, due to job restructuring, Peter change his job. He started to work in his friend's company. His friends and his new co-workers knew that he had a drinking problem which they did not at first address. However, as his friend realised that their customers could see that Peter was intoxicated, he held an intervention. Rather than firing Peter, he started to help Peter save face by excluding him from situations where he might create embarrassing encounters. Peter was no longer allowed to participate in front-stage action with customers, but stayed backstage in situations that required social interaction.

When I started as a brickie (…) my friend said: “you give a damn about talking to customers today… smells today”. He rebuked me but he never said that I shouldn't drink at work (…) I stayed in the car until he had talked with the customers so that I could go out and yes, dig, yes, drive plates or stone floor or whatever we were doing.

We may argue that by protecting Peter and his customers from embarrassing encounters, the employer protected also his own and his firm's reputation. It was a strategy to avoid not only Peter's stigmatisation but also the stigmatisation of his business. Thus, the act of allowing Peter to save face by having him work backstage was “beneficial” for both Peter and his employer.

Little by little Peter's drinking reached a point that his body could no longer endure the effects of drinking. Then he accepted that he no longer could perform a sober self and treat his behaviour as normal, and he wanted to change. He openly acknowledged his drinking problem to his boss and went to treatment to seek help:

When my body [could not take it anymore], I went and talked to my boss and said that “I have a severe alcohol problem”. I quit and then I went straight to social services and asked for help.

## Discussion and conclusions

Our analysis shows how people who struggle with heavy drinking and use drugs at work develop various strategies to make their action appear as normal as possible so that their substance use does not lead to embarrassing and stigmatising situations. On the one hand, by using various substances at work they hide the withdrawal symptoms their heavy alcohol and drug use causes (Kemp & Neale, 2005). On the other hand, they sense that substance use enabled them to perform their work tasks. In this way, substance is a paradoxical tool in performing normalcy.

As work not only provided the participants with material and economic benefits, but also enhanced their self-esteem and helped them to keep their daily routines (Cebulla et al., 2004), our participants developed various strategies to continue working, such as hiding their substance use or minimising its effects on their behaviour and physical appearance. Richard and Johanna's life stories exemplify how the participants perceived that if their heavy substance use was discovered then they would lose face – and feel embarrassed – to such an extent that they would no longer be able to continue working at the same workplace. However, Peter’s life story demonstrates how losing face because of substance use does not always lead to getting fired from work if there is a possibility to redefine the work role. This also exemplifies how employers at times can deny liability for their employees’ heavy substance use (Buvik et al., 2018).

The study also shows how dependency on legal or illegal substances may lead to different challenges when you protect yourself from losing face in front of your colleagues. Obtaining alcohol is an easy process while daily acquisition of heroin is a difficult, time-consuming, and unpredictable process that might result in absence from work. This means that people with heavy drug use need to develop strategies to legitimise and normalise these irregularities by making them appear to be caused by illness, for example, like Johanna did. Using alcohol at work is also less challenging than using drugs. While Richard and Peter could successfully hide their drinking by using a “water bottle” or “coffee thermos” filled with alcohol, Johanna needed to make up several excuses to go to the bathroom to inject.

The expectations to perform normality make the working lives of people who are heavy substance users challenging. Our results demonstrate that our participants were able to maintain a facade of normality and avoid stigmatisation for several years if they managed to deal with their withdrawal symptoms constructively, appeared professional, were willing to change their workplace frequently, or had good stabilised circumstances outside work that prevented their substance use from getting out of hand. The results also highlight that our participants’ strategies for maintaining normality were constantly at risk of failure, and when their performances of normality failed, they lost face and they felt embarrassed. This led to a process in which they were labelled as untrustworthy and they were fired or assigned to taking care of tasks in which the maintenance of normality did not play an important role (Neale et al., 2011).

This study also demonstrates how the participants’ performance of normality varied depending on their main audience. While Richard considered his bosses were the main audience, Johanna assigned this role to her co-workers, and Peter's main audience in the end consisted of customers. Richard could perform his normality at work without the need for a backstage: he kept his withdrawal symptoms at bay with alcohol that he could consume openly in a disguised form. By contrast, Johanna's constant intravenous heroin use would not have withstood the light of day, so her performance of normality required different kinds of backstages: e.g. the bathroom, seeking a place where she could be alone, and directing the interlocutor onto the front stage. Intriguingly, Richard and Johanna were less concerned about their performances towards customers, perhaps because customers come and go but bosses and co-workers are there as long-term witnesses. However, as Peter was allowed to continue to stay at work even after the boss and co-workers became aware of his working under the influence of alcohol, client work was constructed as a front stage from which he had to stay away.

The study has some limitations. As these life stories were produced in face-to-face interviews – interaction situations where interacting parties do face-work and maintain normality – this may have affected the results. The participants may have represented their strategies to produce normality at work by excluding or mystifying those strategies which show them in a negative light, by dramatising or overemphasising those strategies that show them in a positive light, and by forgetting those strategies that come too near the strategies people usually use in everyday life to produce normality. Yet, as most of the participants do not currently struggle with heavy substance use and as their representations of the strategies we have described above deal with their more or less distant past, we may assume that they have gained social distance from them. This may have helped them to narrate their stories honestly and truthfully, even if the events felt embarrassing, stigmatising, and painful when they happened. Moreover, as our interviews belong to the genre of research interviews that encourage the participants to speak about their profoundly private experiences in a confessional mode (Atkinson & Silverman, 1997), the expectation that these interviews provide a secure space for face-work may have lessened the participants’ need to mystify their past.

As our results enhance understanding of the strategies the substance users rely on at work to make their heavy substance use appear normal, they provide resources for people who are responsible for workplace substance use policies to identify problem users for referral to treatment. The results also provide important knowledge for planning work-based interventions for heavy substance use. Moreover, the results facilitate the development of strategies to address substance use problems at work in a non-stigmatising manner and with an understanding that the continuation of work is an important condition for recovery.
